# Cultural interventions to treat addictions in Indigenous populations: findings from a scoping study

**DOI:** 10.1186/1747-597X-9-34

**Published:** 2014-09-01

**Authors:** Margo Rowan, Nancy Poole, Beverley Shea, Joseph P Gone, David Mykota, Marwa Farag, Carol Hopkins, Laura Hall, Christopher Mushquash, Colleen Dell

**Affiliations:** 1 Department of Sociology, University of Saskatchewan, 1109 – 9 Campus Drive, Saskatoon, SK S7N 5A5, Canada; 2 British Columbia Centre of Excellence for Women’s Health, E311-4500 Oak St, Box 48, Vancouver, BC V6H 3N1, Canada; 3 Bruyère Research Institute, 85 Primrose Avenue, Ottawa, ON K1R 6M1, Canada; 4 Department of Psychology, University of Michigan, 530 Church Street, Ann Arbor, MI 48109-1043, USA; 5 Department of Educational Psychology and Special Education, College of Education, University of Saskatchewan, 28 Campus Drive, Saskatoon, SK S7N 0X1, Canada; 6 School of Public Health, University of Saskatchewan, 107 Wiggins Road, Room 2705, RUH Saskatoon, SK S7N 5ES, Canada; 7 National Native Addictions Partnership Foundation Inc, Satellite Office 303 East River Road, Muncey, ON N0L 1Y0, Canada; 8 Department of Psychology and Northern Ontario School of Medicine, Lakehead University, 955 Oliver Rd, Thunder Bay, ON P7B 5E1, Canada

**Keywords:** First Nations, Cultural interventions, Addictions, Indigenous, Treatment interventions

## Abstract

**Background:**

Cultural interventions offer the hope and promise of healing from addictions for Indigenous people.^a^ However, there are few published studies specifically examining the type and impact of these interventions. Positioned within the *Honouring Our Strengths: Culture as Intervention* project, a scoping study was conducted to describe what is known about the characteristics of culture-based programs and to examine the outcomes collected and effects of these interventions on wellness.

**Methods:**

This review followed established methods for scoping studies, including a final stage of consultation with stakeholders. The data search and extraction were also guided by the “PICO” (Patient/population, Intervention, Comparison, and Outcome) method, for which we defined each element, but did *not* require direct comparisons between treatment and control groups. Twelve databases from the scientific literature and 13 databases from the grey literature were searched up to October 26, 2012.

**Results:**

The search strategy yielded 4,518 articles. Nineteen studies were included from the United States (58%) and Canada (42%), that involved residential programs (58%), and all (100%) integrated Western and culture-based treatment services. Seventeen types of cultural interventions were found, with sweat lodge ceremonies the most commonly (68%) enacted. Study samples ranged from 11 to 2,685 clients. Just over half of studies involved quasi-experimental designs (53%). Most articles (90%) measured physical wellness, with fewer (37%) examining spiritual health. Results show benefits in all areas of wellness, particularly by reducing or eliminating substance use problems in 74% of studies.

**Conclusions:**

Evidence from this scoping study suggests that the culture-based interventions used in addictions treatment for Indigenous people are beneficial to help improve client functioning in all areas of wellness. There is a need for well-designed studies to address the question of best relational or contextual fit of cultural practices given a particular place, time, and population group. Addiction researchers and treatment providers are encouraged to work together to make further inroads into expanding the study of culture-based interventions from multiple perspectives and locations.

## Background

The hope and promise of healing from addictions for Indigenous people are rooted in cultural interventions. From sweat lodges [1,2] to traditional teachings [3,4], these regionally diverse interventions are commonly located within the context of Indigenous treatment programs and integrated into existing treatment practices [5]. They are led by individuals who are sanctioned and recognized by traditional teachers, community members, and spiritual beings to facilitate cultural activities [6,7]. For example, in Canada, the 56 National Native Alcohol and Drug Abuse Programs and nine Youth Solvent Addiction Program treatment centres emphasize that Indigenous traditional culture is vital for client healing and wellness [8]. Both programs run under the auspices of First Nations communities and support a network of residential treatment and community prevention programs informed by Indigenous spirituality and origin stories.

Cultural interventions address wellness in a holistic sense, in contrast to Western biomedical approaches that focus on the absence of disease and imply mind-body separation in treating illness such as addictions [9,10]. Key to understanding the benefit of culturally-focused treatment is recognizing the meaning of Indigenous wellness, which is understood as one of a harmonious relationship within the whole person, including mind, body, emotion, and spirit [11-13]. Wellbeing and health emerge from a holistic worldview that emphasizes balance among one’s tradition, culture, language, and community. Szlemko et al. [10] support this notion and suggest that for treatment to be effective it is important to consider the whole person rather than only their physical or mental health.

There are few published studies (i.e., meta-analyses, literature summaries, scoping, or systematic reviews) specifically examining the type and impact of cultural interventions to treat addictions in Indigenous populations, especially with relevance to First Nations of Canada. Many reviews have focused on health education or prevention of substance use problems in Native Americans [14-17]. Some have examined the treatment literature, but have focused on broad populations, such as racial and ethnic minorities [18] or young people [19]. Conversely, others have narrowed their search to specific populations of interest such as Native Hawaiians [20], Hispanic adolescents [21], African Americans [22], or Australian Aboriginals [23-26]. A few reviews have focused on interventions to treat Indigenous people, but these cited interventions are not holistically or culturally-based [27,28]. One literature review considered evidence-based practice in Native American mental health service delivery, but deliberately excluded treatments that targeted substance use [29].

Four relevant literature reviews offer some insight into cultural interventions used, outcomes measured and/or the quality of the research. An early study by Brady [30] involved a review of “comparative material” from the United States, Canada, and Australia on cultural treatments for alcohol addictions in Indigenous people. She found that studies were plagued with poor methodology and lacked clarity about what was actually involved in treatment. Abbott [31] reviewed 10 studies on traditional and Western healing practices for alcohol treatment in Indigenous populations in the United States between 1962-1996. These studies described prevalence data, and the healing practices and Western treatment interventions being implemented, yet the reviewers noted a lack of randomized control outcome studies. Another seven studies from 1970-1989, focused largely on measuring reduction in alcohol consumption, with a notable absence of measuring spiritual and mental functioning. Dell et al. [9] conducted a systematic review of articles published in the Canadian Journal of Psychiatry from February 1998 to June 2008, augmented with a review of Canadian and international literatures on treatment and healing of Aboriginal people for mental health and substance use-related issues. In the 12 selected articles, the authors found a significant gap in understanding and practice between Western psychiatric and Aboriginal culture-based treatment in three areas: connection with self, community, and political context. Finally, Greenfield and Venner [32] conducted a systematic review of the literature from 1965-2011 on substance use disorder treatments for American Indians and Alaska Natives (AI/ANs). Results from twenty-four studies indicated that earlier ones (1968-1997) lacked cultural interventions and took the form of AI/AN counselors and language interpreters. Clinical ratings of improvement were made by treatment staff or community members. Traditional healing approaches were more prevalent in later studies (2000-2011), which also employed formal assessment measures. This shift was viewed as bringing treatment outcomes closer to the AI/ANs’ worldviews.

Although these studies appear relevant to understanding the literature about Indigenous cultural interventions, none explained how information from studies was extracted. There were no details on the screening method or whether multiple reviewers were used to enhance validity of the inclusion or extraction process. Neither Brady [30] nor Abbott [31] listed their inclusion or exclusion criteria so it is unclear exactly which criteria were used to select their studies. While both Dell et al. [9], and Greenfield and Venner [32] listed these criteria, neither focused exclusively on studies with cultural interventions. Neither Brady [30] nor Dell et al. [9] provided a table or summary of the literature reviewed, but rather weaved the information purposefully into narrative discussions to support their ideas. For example, Dell et al. [9] blended literature findings with case study stories to compare and contrast Western and Aboriginal treatment approaches. Finally, Greenfield and Venner [32] focused on historical trends, and while they considered the types of outcomes collected, they did not analyze whether these outcomes focused on different aspects of wellness.

In this article, we report on a scoping study of the literature that explores the use of cultural interventions to treat addictions in Indigenous populations. The purpose of this review is to systematically describe what is known about the characteristics of cultural programs and interventions and to examine the outcomes collected and effects of cultural interventions on wellness. Importantly, the method used for this scoping study draws on evidence described in peer-reviewed and grey literatures. The authors understand that Indigenous epistemologies and other forms of evidence offer additional, and equally important ways of understanding interventions. Our approach is grounded in the concept of Two-Eyed Seeing (*Etuaptmurnk*), whereby Indigenous and Western knowledges are valued and utilized to generate, understand, and find solutions [33]. Furthermore, the scoping study is positioned within the *Honouring Our Strengths: Culture as Intervention* project that builds on our core community-based research team’s history of collectively led projects and aims to create a valid and reliable, culturally-competent instrument to measure the effectiveness of First Nations cultures as an intervention in alcohol and other drug treatments [34].

## Methods

This review followed the design of Arksey and O’Malley [35], enhanced by Levac et al. [36] and involved six stages: Stage 1: Identifying the research question, Stage 2: Identifying relevant studies, Stage 3: Selecting studies, Stage 4: Charting the data, Stage 5: Collating, summarizing and reporting results, and Stage 6: Consulting with stakeholders. The data search and extraction were guided by the “PICO” (Patient/population, Intervention, Comparison, and Outcome) method [37], but we did not require direct comparisons between treatment and control groups. The population included Indigenous people in treatment for problematic substance use or addictions. Cultural interventions were Indigenous spiritual and healing practices or traditions introduced into residential or outpatient treatment centres to help achieve wellness following problematic substance use or addiction. Outcomes included four dimensions of wellness: 1) Spiritual, 2) Physical- Behavioral, 3) Mind- Mental, and 4) Heart- Social and Emotional. Dimensions and their definitions were originally built on the foundational work of two papers [38,39], and later solidified during the project by Elder Jim Dumont after conversations with Treatment Centres. Complete definitions can be found on the *Honouring Our Strengths: Culture as Intervention* website [34].

A librarian scientist helped to develop the PICO criteria. She ran and cross-validated the search strategy with a second librarian. Up to October 26, 2012, 12 databases indexing the scientific literature were searched. These included EBM Reviews (including The Cochrane Library), Global health library, MEDLINE, EMBASE, PsycINFO, Bibliography of Native North Americans, CIHAHL, Social Work Abstracts, Women’s Studies International, Anthropology Plus and Anthropological Literature, Anthropological Index, and CAB direct. Each database was searched using its earliest indexing date. Mesh Headings and free text terms applicable to the PICO criteria were applied separately, or in combinations using the Boolean operators “AND” and “OR”. Under “Population” there were 34 “Heritage or Culture” terms and 24 “Dependence” terms. There were 27 terms under “Interventions” and nine under “Outcome”. Another 13 databases were searched from the grey literature. We supplemented this search with articles identified by or through the research team, relevant websites, hand searching relevant journals, and reference lists of included studies. No restrictions were placed on language. Studies were screened by nine reviewers. An extraction form was developed and pilot-tested to collect detailed information about the background, measurement, and results of each study. Information was then entered into Word and Excel files, integrated, and summarized in display and written format.

## Results

### Yield

**Figure 1:**
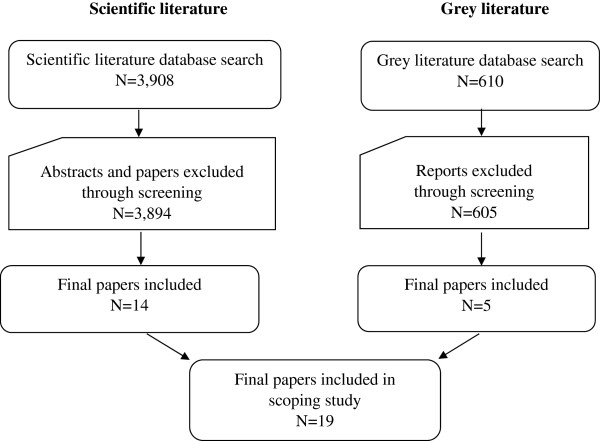
Yield from literature search up to October 26, 2012.

The search strategy yielded 4,518 articles of which 19 studies, involving 5,949 treatment clients, were included in the final review. Fourteen of these were from the scientific literature and five were from the grey literature (see Figure 1). Most often studies were excluded because they were descriptive, anecdotal, or preliminary; did not report or collect outcomes; and/or did not report or include cultural interventions. Decisions about article inclusion or exclusion were resolved through consensus between pairs of reviewers or between an arbiter and a reviewer.

### Characteristics of programs and interventions

Table 1 describes each study by location, type and length of program, and interventions provided.

**Table 1:** Characteristics of scoping study scientific and grey literature: program descriptions

**Author and Location (Country/Region)**	**Program type and length**	
	**Cultural interventions**	**Western interventions**
**Scientific literature**		
Anderson, 1992/CAN/ BC [40]	Community treatment centre: 6 week alcohol addiction program.	
	• Ceremonial practice (some smudging done each morning, but used more by staff than clients).	• Group sessions (the entire client population of six families per 6 week session, meet together 3-5 times a week for half a day. In this "circle," communication, listening and attending are established founded on mutual respect and unconditional positive regard).
	• Land base activities (focus on healing qualities of the physical site).	• Family counseling.
	• Social culture (community and social activities of community suppers, food shopping, chapel services, and recreational pursuits such as fishing and volleyball, helped clients relate as families and neighbors without alcohol).	• Alcoholics Anonymous meetings.
		• Individual and couples counseling sessions and special work with children and young adults.
Boyd-Ball, 2003/US/ Pacific NW [41]	Residential: 8 week alcohol and drug addiction program in study known as Shadow Project. Comparison of (culturally supplemented) Treatment As Usual (TAU) and treatment with family-enhanced intervention.	
	• Sweat lodge.	• Individual therapy.
	• Ceremonial practice (a Welcome Home ceremony involving family and community support- for the family-enhanced intervention; naming ceremony).	• Group therapy.
	• Land base activities (wilderness outings, a Welcome Home ceremony involving family and community support for the family-enhanced intervention).	• 24-hour supervision.
	• Traditional teachings- studied individual tribal histories.	• Psychiatric and psychological services.
	• Singing.	• Assessment and referral.
	• Cultural instruments (drumming).	• Life-skills counseling.
	• Story-telling—used in the family-enhanced intervention only.	• Medical services.
	• Art creation (crafts).	• Education programs.
	• Elders (access to spiritual elders).	• Family programs.
		• Aftercare planning.
Boyd-Ball et al, 2011/US/ Western regions (from 8 States) [42]	Residential: 7 week substance use treatment emphasizing traditional practices at the “WAIT” Center. Post-treatment substance use trajectories were correlated with self-report measure of general American Indian (AI) cultural involvement.	
	• Sweat lodge (“sweats”).	• Family management.
	• Other ceremonial practice (not specified).	
	• Post-treatment social cultural participation (speculation that perhaps adolescents were prepared in treatment for greater involvement in tribal culture & traditions on returning home).	
Dell & Hopkins, 2011/CAN/across Canada [43]	Residential: 4-6 month solvent use program.	
	• Fasting.	• Treatment and support based in resiliency theory.
	• Land base activities (land-based cultural camps).	• Support for development of emotional intelligence, personal wellness care practices, and leadership skills (within a positive psychology framework).
	• Traditional teachings (Elders’ teachings).	
	• Social culture (inclusion of community members in the treatment centers).	
	• Natural foods and medicines (ceremonial feasts).	
	• Elders (Elder guidance).	
Dell et al, 2011/CAN ON [44]	Residential: 12 week 1 h per week, Equine assisted learning (EAL) curriculum added to a 4 month solvent use program at Nimkee NupiGawagan Healing Centre (NNHC).	
	• Land base activities (Equine Assisted Learning programs help make a connection to nature and the horse(s) within a natural environment).	According to Bresette (2009/2010), NNHC offers:
	• Other cultural aspects to the program in addition to the equine therapy includes: Bi-weekly sweats, Welcoming Feasts, Full Moon ceremonies, Memorial Feasts, Spring Releasing ceremony, Spring and Fall Fasting, Youth Naming Ceremonies, Berry picking, Rites of passage ceremonies (i.e., Berry Fast), Pow-wows, Gardening, 1-1 cultural teachings, Traditional healer visits.	• Individual and group counseling therapy.
		• Learning centre and work placements.
		• Nutrition program.
		• Health care.
		• Recreation activities, including attending sporting events.
		• Aftercare planning and follow-up.
Edwards, 2003/US/CA [45]	Residential: 90 day substance use program and 90 day aftercare program, at Friendship House.	
	• Sweat lodge.	• Individual and group counseling.
	• Traditional teachings (the re-traditionalization process teaches clients about Native American values and traditions in classes such as "The Red Road" based on the work of Gene Thin Elk (1993) and "Native American Family Values").	• Co-dependency group work.
	• Singing.	• Alcohol, drug, and HIV/AIDS education.
	• Cultural instruments (drumming).	• Alcoholics Anonymous and Narcotics.
	• Talking circle.	• Education about historical Native American traumas.
	• Social cultural (Friendship House celebrations, personal relationships with the Native American staff members).	
	• Traditional healers (Medicine people).	
Gossage et al, 2003/US/AZ [46]	Prison-based: Sweat lodge ceremony offered to prisoners to treat alcohol addiction.	
	• Sweat lodge.	• Alcohol education.
		• Group psychotherapy.
D’Silva et al, 2011/US/MN [47]	Community-based: 4, 1 hr. individual or group tobacco cessation sessions paired with pharmacotherapy.	
	Culturally modified the American Lung Association’s ‘Freedom from Smoking’ program incorporating:	• Community outreach and education.
	• Traditional teachings on how to use tobacco as a sacred item in ceremonies and offerings. These teachings are designed to help participants understand the difference between sacred tobacco use and commercial tobacco addiction.	• Clinical system referrals.
	• Story-telling – cultural adaptations were made to counseling sessions based on suggestions from key community stakeholders, and included the addition of Ojibwe stories.	• Individual and group counseling.
	• Language (use of Ojibwe language in treatment sessions).	• Access to nicotine replacement therapies (NRT) and prescription medications.
Lowe et al, 2012/US/OK [48]	Community-based: Two types of substance use interventions: 1) Cherokee Talking Circle (CTC), a culturally based, 10, 45 min intervention and 2) Be A Winner/Drug Abuse Resistance Education (DARE), 10, 45 min standard sessions.	
	The Cherokee Talking Circle intervention incorporated:	DARE education program:
	• Language (the manual used both English and Cherokee languages).	• Promotes a school/law partnership approach to substances/ drug education.
	• Talking circle.	
^1^Naquin et al, 2006/US/AK [49]	Residential: Alcohol addiction treatment program within the Ernie Treatment Centre, under the Cook Inlet Tribal Council (CITC) called the Therapeutic Village of Care. Treatment is organized into three phases: Orientation, Stabilization, and Right Living. The length of time in each phase depended on resident’s treatment plan or progress.	
	• Sweat lodge (steam bath similar to an American Indian sweat lodge).	CITC offers:
	• Ceremonial practice (harvesting moose (road killed)).	• Street outreach.
	• Social culture (residential treatment community functions as a large extended family: Members assume the roles of ‘aunties’ and ‘uncles’; mature members teach and mentor other, newer family members and help them reconnect with their family histories and culture by sharing their knowledge of tribal genealogies; staff participate as equals, modeling appropriate family roles and relationships. They also serve as guides, facilitating the healing process through role modeling and participation in, but not control of, the community).	• Case management.
	• Elders (assume traditional role and are a constant reminder to residents of unspoken Native cultural norms).	• Screening and brief intervention.
	• Art creation (carving).	• Assessment and brief treatment.
		• Emergency care and detoxification.
		• Intermediate residential, outpatient and continuing care.
^1^Nebelkopf & Penagos, 2005/US/CA [50]	Residential, Health Centre, and Outpatient: HIV/AIDS, substance use, and mental health programs are offered under the Holistic Native Network (HNN).	
	There were seven projects that comprise the HNN. Four of these projects focus on substance use (Native Youth Circle, FH Healing Circle, Urban Native Youth, and Native Women). The remainder are concerned with mental health or HIV/AIDS. Types of cultural interventions and examples are provided below:	HNN offers:
	• Sweat lodge (monthly gatherings where members of the community where members of the community come together in a spiritual way).	• Residential treatment.
	• Natural foods and medicines (traditional herb consultations).	• Outpatient counseling (individual, group or family counseling).
	• Cultural instruments (drum group).	• Case management.
	• Talking circle.	• Community outreach.
	• Traditional teachings (discuss the Red Road to Recovery).	• Risk-reduction counseling.
	• Art creation (beading class).	• Psychotherapy.
	• Social culture (Pow-wows, barbecues, dinners, ceremonies, give-aways, health fairs and other rituals are planned monthly and with the changing of the seasons).	• Art therapy.
	• Traditional healers (a central component at community events).	• Home visits.
^1^Nebelkopf & Wright, 2011/US/CA [51]	Community-based: Substance use treatment within the Native Men and Native Women Program.	
	The program is one of three described under the Family and Child Guidance Clinic (FCGC) of the Native American Health Center, Holistic System of Care (HSOC) for Native Americans in an Urban Environment. The other two are not of primary interest as they focus on prevention and children’s mental health. The HSOC model includes:	FCGC offers:
		• Individual, group and family counseling.
	• Sweat lodge.	• Care coordination.
	• Ceremonial practice (seasonal ceremonies, smudging).	• Psychological assessment.
	• Traditional teachings (discuss the Red Road to Recovery).	• Screening.
	• Prayer.	• Alcohol and drug prevention programs for youth and adults.
	• Social culture (four-day Gathering of Native Americans (GONA)).	• HIV/AIDS prevention.
	• Story-telling.	• Youth Services program: Drop-in centre, after-school services, tribal athletics, and substance abuse prevention.
	• Talking circle.	
Saylors, 2003/US/CA [52]	Residential: Substance use treatment provided by the Women’s Circle at two Native American Health Centres.	
	Cultural interventions often occur at an individual level, with counselors assessing a client's desire or readiness to work with traditional ways. A counselor's initial clinical assessment contains spiritual/cultural domains that allow him/her to gauge a client's cultural affiliation and identification. This helps direct the development of a treatment plan which may include:	• Psycho-therapeutic practice.
	• Sweat lodge.	• Family and Child Guidance Clinic provides the services of a nurse case manager and perinatal social worker.
	• Singing.	
	• Cultural instruments (drumming).	
	• Natural foods and medicines (herbs and tobacco).	
	• Traditional healers (Native healers from different cultural backgrounds and traditions are brought in for several days at a time to work with clients).	
	• Prayer (some counselors pray with clients at the client's request).	
	• Ceremonial practice (sage, cedar or sweet grass smudges are often incorporated into a counseling session).	
	• Talking circles (held regularly at the clinic for clients and staff).	
Wright et al, 2011/US/CA [53]	Residential and Outpatient: Mental health and substance use treatment at the Native American Health Center (NAHC) using the Holistic System of Care (HSOC) service provision framework.	
	Native American culture is integrated into treatment in the following ways:	HOSC offers:
	• Sweat lodge.	• Treatment (mental health, substance use, medical, and family services).
	• Ceremonial practice (seasonal ceremonies, smudging).	• Prevention (wellness education, positive parenting intervention, mental health promotions, addiction prevention, hepatitis prevention, and HIV/AIDS prevention).
	• Traditional teachings (self-directed learning: Drawing on intertribal similarities, counselors also work with individuals to develop skills and use healing practices that includes individual backgrounds, traditions, practices, and stories).	• Recovery services (employment, housing life skills, and community service (giving back)).
	• Natural foods and medicines (herbs).	• Peer support.
	• Cultural instruments (drumming).	
	• Talking circle.	
	• Social culture (Pow-wows, women’s/men’s/youth societies, GONA, Positive Indian Parenting (OIO)).	
	• Prayer.	
	• Story telling.	
	• Traditional healers (Native healers from different cultural backgrounds and traditions are brought in for several days at a time to work with clients).	
**Grey Literature**		
Bresette, 2009/ 2010/ CAN/ON [54]	Residential: 4 month solvent addictions treatment provided at Nimkee NupiGawagan Healing Centre Inc.	
	• Sweat lodge (bi-weekly, staff sweats).	Centre offers:
	• Fasting ceremony (spring and fall fasting).	• Individual and group counseling therapy.
	• Ceremonial practice (Full Moon ceremonies, Spring Releasing Ceremony, youth naming ceremony, rites of passage ceremonies, smudging. Multicultural and certified staff (Anishnaabe, Haudenosaunee, Lenni-Lenape) accommodate specific cultural and healing experiences).	• Learning centre and work placements.
	• Land base activities (gardening, equine program).	• Nutrition program.
	• Traditional teachings (one to one cultural teachings).	• Health care.
	• Social culture (Pow-wows).	• Recreation.
	• Natural foods and medicines (welcoming feasts, memorial feasts, berry picking).	• Aftercare planning and follow-up.
	• Singing.	• Community education and training.
	• Cultural instruments (drumming).	
	• Prayer.	
	• Language (encourages and reinforces communication in original language).	
	• Traditional healers.	
D’Hondt, no year/CAN/ON [55]	Residential: 21 day cycle substance use treatment cycles at the Centre for Addiction and Mental Health Addiction Program (CAMH).	
	• Ceremonial practice (smudging).	Document lists the following services for pilot program:
	• Cultural instruments (drumming).	• Employment and housing for treatment graduates.
		• Aftercare programs.
		CAMH, in general, offers a variety of services (see: http://www.camh.ca/en/hospital/care_program_and_services/addiction_programs/Documents/3882ABS_brochurestnd.pdf
		Including:
		• Intake and assessment.
		• Individual, couple and family counselling.
		• Talking circles and group work.
		• Telephone counselling.
		• Training, consultation and capacity building.
		• Inpatient and outpatient treatment programs.
		• Referrals.
Kunic, 2009/CAN/across country [56]	Prison-based: Aboriginal Offender Substance Abuse Program (AOSAP) offered to male offenders involving four modules and 65 sessions.	
	• Sweat lodge.	AOSAP offers contemporary best-practices in substance use treatment, such as cognitive-behaviourism, social learning theory, and relapse prevention.
	• Ceremonial practice (sacred sweat ceremonies plus other ‘traditional ceremonies’ relevant to the place in which they are conducted, however no detail as to what these ceremonies are is provided).	
	• Traditional teachings (particularly within the Modules 1 and 4, e.g., power of the circle of wellness).	
	• Natural foods and medicines (sacred medicines introduced in Module 4).	
	• Social culture (The Western Door (Module 3), which is 14 sessions in length, focuses on the history of consequences and the impact of substance use within Aboriginal communities. It also explores the devastating effects of substance use on Aboriginal individuals, families, and communities, and how changing individual behavior can result in the restoration of health, pride and culture). Module 2- Aboriginal spiritual engagement is facilitated through the introduction and exploration of the impact of trauma and how substance use was, and still is, a means by which Aboriginal people tried/try to cope with its effects).	
	• Talking circle.	
McConnery & Dumont, 2010/CAN/QC [57]	Residential: 5 week alcohol and substance addiction treatment program at Wanaki Centre.	
	• Sweat lodge.	• Cognitive-behavioural therapy.
	• Ceremonial practice (letting go ceremony after Sweat lodge. Have a Closing of the Sacred Fire ceremony with the Elder that provides closure for the entire treatment cycle. Smudging daily).	• Life skills training.
	• Land base activities (teaching and experiences that build connections to creation/nature- clients go in the forest to collect cedar and balsam for the Sweat Lodge ceremony. Spending time in the woods with an Elder).	
	• Traditional teachings (delivered by an Elder: Sacred Fire, Pipe Keeper, four medicines, blessing of the water, teachings for women such as moon time and women’s dress, teaching of the lodge. Also have traditional Algonquin teachings. Adhere to the philosophy of 1) Red Road – involves a strict code of conduct and ethics, the foundation being respect for oneself and for other people and the environment in all its forms. 2) Medicine Wheel: Mental, emotional, spiritual, and physical).	
	• Social culture.	
	• Natural foods and medicines (have a cooking workshop to make traditional foods. A traditional meal is offered to clients, staff and guests at the graduation ceremony).	
	• Singing (songs are used with the Blessing of the Water teaching).	
	• Cultural instruments (drumming is used with the Blessing of the Water teaching).	
	• Language (use of Algonquin language).	
	• Talking circle (Sharing circle—the Eagle Feather is used here. Healing circles lead by Elder).	
	• Elders (Elders from the community and abroad deliver the teachings and traditional components of the program).	
	• Art creation (Grieving collage made of pictures cut out of magazines, representing images that touched them personally and they present their collage to the group. Create a family genogram showing family members who suffered from addictions. Make dream catchers and grieving bags).	
	• Prayer (daily).	
^1^The Tsow Tun Le Lum Society, no year/ CAN/BC [58]	Residential: 42 day alcohol and drug treatment program provided at the Tsow Tun Le Lum Society.	
	• Sweat lodge.	• Client outreach.
	• Ceremonial practice (traditional food burnings at least twice per year).	• Community networking and development.
	• Land base activities (spring-fed pond for traditional cleansing).	• AA and NA meetings.
	• Singing.	• Aftercare.
	• Dancing.	
	• Cultural instruments.	
	• Elders (Elders lead the morning “Spiritual Room” session that begins each program day. Healthy reconnection to “being Indian” is the goal of the unique Elder component).	
	• Prayer.	

^1^ Focused on part of study relevant to scoping study only.

### Location

All of the 19 studies were from the United States (58%) and Canada (42%). Most studies (79%) were localized within a community or communities of one state or province, particularly California or Ontario. In some cases, clients were referred from outside the community. For example, clients at the Friendship House Association of American Indians in San Francisco were referred by Indian health programs in six other states [45]. Another program located in the Pacific Northwest, drew young clients from eight states across the Western region of the US [41,42]. There were two national level studies. Dell and Hopkins [43] studied treatment practices and outcome data from nine Youth Solvent Addiction Program sites across Canada. Similarly, Kunic [56] evaluated the effects of a national program entitled the Aboriginal Offender Substance Abuse Program sponsored by Correctional Services Canada. Two treatment facilities served as sites for multiple studies included in this review, one located in the Pacific Northwest [41,42] and the other based in San Francisco [45,50-53].

### Type and length of treatment programs

For the most part, studies involved residential programs (58%) of varying lengths. For example, one of the shortest residential programs was described by Boyd-Ball [42] as a 7 week treatment for young, tribally enrolled substance users, involving cultural interventions and family management. In contrast, Dell and Hopkins [43] studied a 4-6 month residential program for young solvent users that combined culture-based interventions with Western-based, positive psychology programming. Other programs were community-based (21%), prison-based (11%), or offered at minimum a combination of residential and outpatient services (11%). Most focused on addiction treatment (63%), three concentrated on treatment for alcohol, and three for solvent use. Another study by D’Silva et al. [47] focused exclusively on tobacco cessation.

### Interventions provided

All studies (100%) involved integrative treatment programs, meaning that the site(s) offered the client and possibly his/her family, Western-based assessment, education, counseling, treatment, and/or aftercare services alongside cultural and traditional services. For example, Boyd-Ball [41] studied the Shadow Project, an 8 week residential program in which the treatment as usual (TAU) offered Western services such as group therapy and life-skills counselling. TAU was supplemented by traditional cultural interventions, such as sweat lodge ceremonies and access to spiritual Elders. The alternate intervention included TAU plus family-enhanced involvement. More recently, Nebelkopf and Wright [51] and Wright et al. [53] applied a Holistic System of Care for Native Americans in an urban setting. This was a community-focused intervention involving Western and culturally-based prevention, treatment and recovery programs.

**Figure 2:**
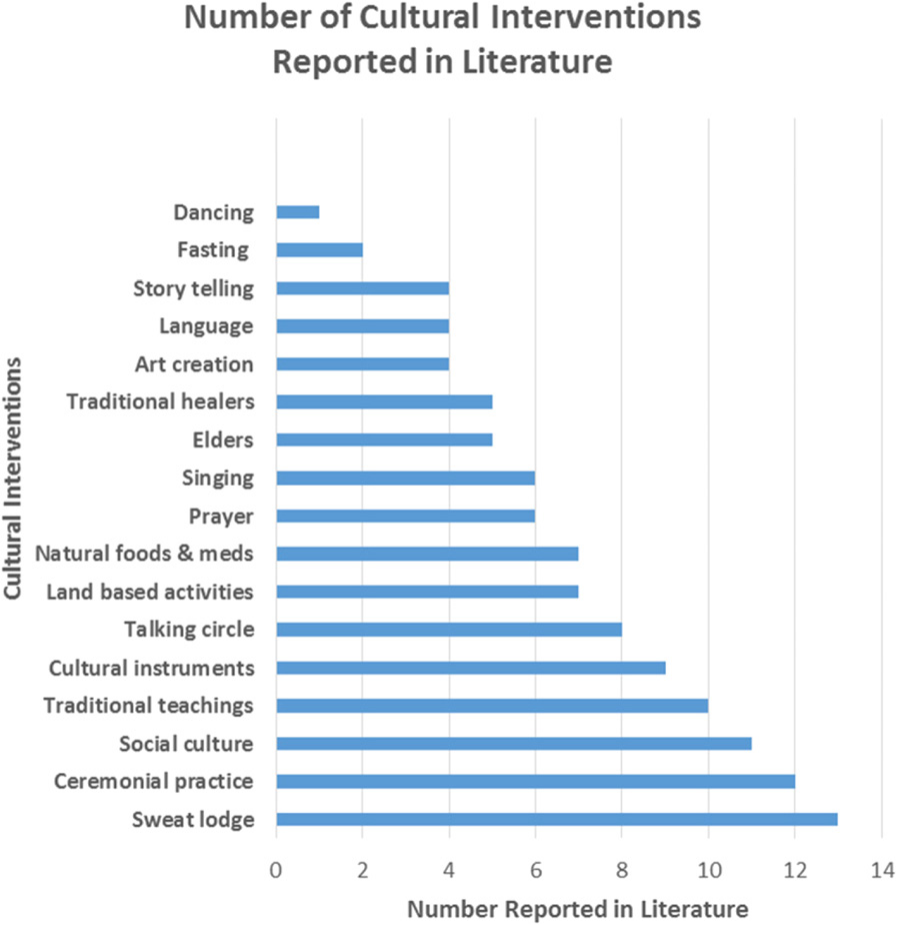
Number and type of cultural interventions reported in the literature.

Seventeen different Indigenous cultural interventions were reported in the literature (see Figure 2). The number of cultural interventions ranged from 1-13 per study, with a mean of six interventions. There were eight studies with 1-5 cultural interventions; nine with 6-10; and two with 11-13. Most studies (68%) included sweat lodge ceremonies, as highlighted in Gossage et al. [46]. Also commonly reported were ceremonial practices (63%), such as sage, cedar, or sweet grass smudges [40,52]; social cultural activities (58%), as emphasized in Naquin et al.’s therapeutic community that treats clients as family [49]; and/or traditional teachings (53%), such as classes in the “Red Road” [45]. Dancing was the least common main intervention reported in only one study, although it was sometimes incorporated within other interventions such as sweat lodge ceremonies.

### Study samples, designs and methods

Table 2 summarizes the samples, designs, and methods of the studies included.

**Table 2:** Study samples, designs, and methods

**Study**	**Samples**	**Designs**	**Methods**
**Scientific Literature**			
Anderson, 1992 [40]	63 clients: 39 clients were 1-2 years out of treatment; another 24 clients had gone through the program less than a year before.	Qualitative: Ethnographic study whereby the author and another researcher resided in the community for two months. They observed and participated in the 6 week program.	Mixed methods—Interviews with clients post-treatment (open-ended, face-to-face, frequently with multiple interviews of the same persons and usually in family contexts), observations of treatment, personal testimonies and materials written by staff and clients.
Boyd-Ball, 2003 [41]	57 clients (and their families): 31 males; 26 females; mean age: 16 years old.	Quasi-experimental: Non-equivalent control group. Comparison of (culturally supplemented) Treatment As Usual (TAU) and TAU with culturally and historically family-enhanced intervention.	Surveys—All clients (and their families) followed up and assessed monthly for 11 months from the day they left treatment. Follow-ups were also done the third and final year of the study.
Boyd-Ball et al, 2011 [42]	57 clients (and their families): 32 males; 25 females; mean age: 16 years old.	Quasi-experimental: Time-series. Post-treatment substance use trajectories were correlated with self-report measure of general American Indian (AI) cultural involvement.	Mixed methods—Surveys, interviews, and observation. Data were collected in three waves: baseline, monthly for 11 months post treatment, and at exit interview 12 months following treatment.
Dell & Hopkins, 2011 [43]	154 youth.	Quasi-experimental: Time-series data used to provide insights into the Youth Solvent Abuse Program (YSAP) treatment program outcomes.	Surveys at 3, 6, 9 and 12 month intervals.
Dell et al, 2011 [44]	15 youth (two intakes of program): 7 males; 8 females; mean age: 14-15 years old; 6 treatment staff.	Qualitative: Exploratory, phenomenology study to understand the experiences of First Nations and Inuit youth participating in an Equine-Assisted Learning (EAL) program as part of their healing from solvent addiction while in a residential Treatment Centre.	Mixed methods—Interviews with youth and staff held during last week of program (semi-structured, face-to-face), researcher observations, written reflections by researchers, program facilitators and staff of EAL program, and journal responses by youth during the program.
D’Silva et al, 2011 [47]	317 adults.	Quasi-experimental: Time-series. A single-group design involving an evaluation of a culturally specific curriculum for tobacco dependence treatment.	Mixed methods—Self-reported tobacco use assessed at baseline, exit, and follow-up included current smoking behaviours and quit attempts; seven-day point-prevalence abstinence measured at exit and follow-up; and pharmacotherapy data obtained from program records.
Edwards, 2003 [45]	12 adults: 6 males; 6 females; age range: 23-51 years old.	Qualitative: Grounded theory study to understand and document the experience of substance use recovery from the perspective of the Native Americans in treatment.	Interviews—single, face-to-face, conducted after completion of the 90 day residential substance use treatment program.
Gossage et al, 2003 [46]	190 males: mean age: 30 years old. The sample was divided into two groups: IPsFU and IPsNFU. The size of each group varied by stage of measurement but generally there were equal numbers in both groups.	Quasi-experimental: Time-series and comparison between inmate/patients (IPs) who were followed-up (IPsFU) vs. those not followed-up (IPsNFU) to advance current knowledge about the efficacy of Sweat Lodge Ceremony. It is unclear what follow-up entailed.	Surveys—Four different surveys used at distinct stages: baseline; multiple times after sweat lodge experiences; and 3 and 9 months after release.
Lowe et al 2012 [48]	179 students: Intervention #1—92 students: 59 males; 33 females; mean age: 17 years old; Intervention #2—87 students; 44 males; 43 females; mean age: 16 years old.	Quasi-experimental: Non-equivalent control group. Two condition design: 1) Cherokee Talking Circle (CTC) and 2) Be A Winner/Drug Abuse Resistance Education (SE).	Surveys—Three instruments used to make comparisons at pre-intervention, immediate post-intervention, and 90 day post-intervention.
^2^Naquin et al, 2006 [49]	399 clients: 203 males; 196 females.	Pre-experimental: One-shot case study examining resident engagement with treatment process and outcomes at a single Treatment Centre.	Mixed methods—Time in treatment/retention rates compared to earlier years and national averages and Surveys—post-treatment perception of care; 6 month follow-up of level of employment and use of alcohol.
^1^Nebelkopf & Penagos, 2005 [50]	45 individuals: 39 males; 5 females; 1 transgender.	Pre-experimental: One group pretest-posttest examining changes in clients’ quality of life as a result of services received through the Holistic Native Network.	Survey—Pre-post survey at baseline and 3 months after care.
^1^Nebelkopf & Wright, 2011 [51]	490 adults: 142 males; 348 females.	Pre-experimental: One group pretest-posttest involving adult substance users to assess whether the Holistic System of Care for Native Americans is a viable model of treatment.	Survey—Pre-post survey at baseline and 6 months after care.
Saylors, 2003 [52]	742 females.	Pre-experimental: One group pretest-posttest to assess lessons learned and impact of the Substance Abuse Treatment Women’s Circle on clients.	Survey—Pre-post survey at baseline and 12 months follow-up.
Wright et al, 2011 [53]	490 participants: 142 male; 348 females; mean age: 36 years old.	Pre-experimental: One group pretest-posttest to assess preliminary outcome findings of substance abuse outpatient and residential treatment services for urban American Indians and Alaskan Natives under the Holistic System of Care model of treatment.	Survey—Pre-post survey at baseline and 6 months after care.
**Grey Literature**			
Bresette, 2009/ 2010 [54]	27 clients: 9 males; 18 females, mean age: 16 years old.	Quasi-experimental: Time-series to execute an impact evaluation of the Nimkee NupiGawagan Health Centre Inc. pilot project involving treatment for youth, families, and their communities who suffer from solvent addiction.	Surveys—Pre-post survey at 3 and 6 month follow-up.
D’Hondt, no year [55]	12 clients.	Quasi-experimental: Time-series to evaluate a pilot residential substance use treatment program at the Centre for Addiction and Mental Health.	Mixed methods—Focus groups, and interviews and surveys at baseline, treatment completion, and follow-up.
Kunic, 2009 [56]	2,685 males.	Pre-experimental: One-shot multiple case studies comparing treatment outcomes among three treatment groups: 1) the Aboriginal Offenders Substance Abuse Program (ASOP), 2) the National Substance Abuse Program—High Intensity (NSAP-H) or 3) Moderate Intensity (NSAP-M).	Mixed methods—Comparison of post-release outcomes over an 18 month follow-up period among three treatment groups: Biochemical markers—urinalysis for evidence of drug use and program records—type of release and revocation.
McConnery & Dumont, 2010 [57]	15 clients: 10 males; 5 females.	Quasi-experimental: Time series to study the impact of an integrated addictions treatment program at Wanaki Treatment Centre.	Survey interviews—Repeated measures surveys (in person and by telephone) at baseline, end of treatment, and 3 and 6 months post treatment.
The Tsow Tun Le Lum Society, no year provided [58]	11 clients: 6 males; 5 females.	Quasi-experimental: Time series to assess the integrated alcohol and drug treatment program provided at the Tso Tun Le Lum Society.	Survey interviews—At admission, completion of program and 3 months post treatment.

^2^Focused on part of study relevant to scoping study only.

### Samples

Samples ranged from 11 to 2,685 clients who participated in a culture-based treatment program for problematic substance use. Most studies included both male and female participants. Two studies involved solely male clients attending a prison-based intervention program [46,56] and one included solely female clients of Native American Health Centres [52]. Another three focused on adult clients: Two involved youth and one evaluated students. In studies that reported the average age of clients, the mean age range was from 14 to 36 years old.

### Designs

Many research designs were utilized; none were true experimental designs. Just over half (53%) of studies involved quasi-experimental designs. Commonly within these designs researchers collected data from clients before treatment or at baseline and then reassessed at multiple points during or after treatment. For example, Gossage et al. [46] collected time-series survey data from 190 males enrolled in a jail-based alcohol treatment program at pretest; multiple times after sweat lodge experiences; and 3 and 9 months after release. Two other quasi-experimental studies involved a non-equivalent control group design, comparing the effectiveness of cultural and Western interventions. To illustrate, Lowe et al.’s [48] two condition design compared survey scores in years two and three from 87 students of the “Be a Winner/Drug Resistance Education” and 92 students in the traditional Cherokee Talking Circle group. Pre-experimental designs were employed by roughly a third (32%) of studies. These designs commonly assessed one group before and evaluated the same group 3, 6, or 12 months after treatment. For example, Saylors [52] used this design to survey changes in 742 females engaged in residential treatment at baseline and at 12 month follow-up. Finally, three studies (16%) used qualitative designs employing ethnographic, phenomenological, or grounded theory approaches.

## Methods

A total of 16 studies used surveys; nine of these studies (56%) developed in-house surveys and seven studies (44%) incorporated a total of 14 standardized instruments to measure mostly self-reported physical and/or emotional aspects of wellness (see Table 3). Only Boyd-Ball et al. [42] reported reliability scores using test-retest scores. There were six standardized surveys identified to measure alcohol or drug use: 1) Behavioral and Symptom Identification Scale-32 (Basis-32) [55], 2) Global Assessment of Individual Needs—Quick (GAIN-Q) [48], 3) The Government Performance Results Act (GPRA) Tool [51,53], 4) Form 90-DI: A Structured Assessment Interview for Drinking and Related Behaviors [42], 5) Alcohol Dependence Scale (ADS) [56], and 6) Drug Abuse Screening Test (DAST) [56]. The American Indian Cultural Involvement Index [42] and the Cherokee Self-Reliance Questionnaire [48] were the only instruments that were oriented to Indigenous culture.

**Table 3:** List and type of standardized surveys

**Survey [study where used]**	**What it measures**	**Administration: self-report = SR; caregiver = CG**
Behavior and Symptom Identification Scale-32 (Basis-32) [55].	Relationship to self and others, depression and anxiety, daily living skills, impulsive and addictive behaviors, and psychosis.	SR
Global Assessment of Individual Needs – Quick GAIN-Q [48].	Four major scales – General Life Problem Index (GLPI), Internal Behavior Scale (IBS), External Behavior Scale (EBS), and Substance Problem Scale (SPS).	SR
The Cherokee Self-Reliance Questionnaire [48].	Presence of Cherokee self-reliance.	SR
The Government Performance Results Act (GPRA) tool [51,53].	Demographics; drug and alcohol use; family and living conditions; education, employment, and income; crime and criminal justice; mental and physical health problems; treatment/recovery; and social connectedness.	SR
Child Behavioral Checklist (CBCL) Behavioral and Emotional Rating Scale [51].	Behavioral and emotional problems in children.	CG
Caregiver Strain Questionnaire (CGSQ) [51].	Strain such as feelings of anger and resentment about the child, disruption of family and community life, and caregiver feelings of worry, quilt, and fatigue.	CG
Columbia Impairment Scale (CIS) [51].	Global impairment for youth.	CG
Behavioral and Emotional Rating Scale (BERS-2 Caregiver) [51].	Interpersonal, intrapersonal, family, affective, school, and career strengths.	CG
Quality of Life (QOL) Survey [50].	Gender, ethnicity, education, presence of an AIDS diagnosis, and quality of life.	SR
Form 90-DI: A Structured Assessment Interview for Drinking and Related Behaviors [42].	Alcohol consumption and other related problems.	SR
The American Indian Cultural Involvement Index (AICI) [42].	Composite score based on two measures: 1) a child ethnic identity score and 2) count of traditional values practiced or believed.	SR
Alcohol severity ratings on the Alcohol Dependence Scale (ADS) Problems Related to Drinking Scale [56].	Alcohol dependence syndrome.	SR
The extent of problems related to drinking as measured by the Problems Related to Drinking Scale (PRD) [56].	Alcohol-related problems.	SR
The drug severity ratings on the Drug Abuse Screening Test (DAST) [56].	Severity of problems associated with drug use.	SR

### Wellness outcomes collected and main results

Table 4 summarizes the outcomes collected and main results from the studies identified in the scoping study.

**Table 4:** Wellness outcomes and main results

**Study**	**Wellness outcomes**				**Main results**
	**Spiritual**	**Mental**	**Emotional**	**Physical**	
	**Scientific literature**				
Anderson, 1992 [40]			√	√	• 1/3-1/2 of clients maintained sobriety for at least 1 year post treatment.
					• Clients established follow-up circles in their own community and those involved “do much better and feel more hopeful than those that are not” (p.11).
Boyd-Ball, 2003 [41]			√	√	• Family-enhanced group perceived high level of support of family members (94.2%) and nonfamily adults (90.6%) and positive peer support (66%).
					• % of days abstinent from substance use from month 1 to 12 was high for both (culturally supplemented) treatment as usual and family-enhanced intervention groups, ranging from 80-100% days abstinent.
					• The highest gain in abstinence was from month 1 to 2 for both groups.
Boyd-Ball et al, 2011 [42]	√	√	√	√	• At 1 year follow-up: 23% relapsed into regular substance use; 77% showed low levels of substance use.
					• Post-treatment substance use trajectories indicated that membership in the relapser’s group showed less engagement in traditional cultural practices and identification with their American culture (mean = -.24) than those classified in the abstainers group (mean = .17).
Dell & Hopkins, 2011 [43]		√		√	• Half of the youth (49.62%) reported a completely abstinent lifestyle in 90 days following exit from the program and half of these youth (51%) reported to not have the urge to misuse volatile or other substances during this time.
					• At 6 months follow-up, 74% reported not using volatile or other substances and 68% of these reported not having to resist drug use.
					• More than half of youth who completed the program (54.2%) reported attending school at 3 month follow-up and at 6 months this rate increased to 83.64%.
Dell et al, 2011 [44]	√	√			• Participating in the Equine-Assisted Learning program provided a culturally relevant space for youth and thus was beneficial to their healing in the program.
					• Three main themes explained the healing experience: spiritual exchange (calm presence, being in the moment, meaningful connection to the horse), complementary communication (ability to communicate with horse beyond verbal commands and helped with patience and leadership in communicating with others), and authentic occurrence (females showed compassion for pregnant mares and foals, interacting with horse let them experience healthy touching and expressing affection).
D’Silva et al, 2011 [47]				√	• 63% of participants completed the program.
					• Upon completion, almost 1/3 of participants self-reported 7 day abstinence.
					• Of those reached at follow-up, 47% reported abstinence at 90 days.
					• The smoking quit rate was 21.8%.
					• Continuing smokers cut their daily smoking by half (from 17-9 cigarettes).
					• 88% reported an increase in self-efficacy for their next quit.
					• 44% planned to quit within 30 days.
Edwards, 2003 [45]	√	√	√		• 73 transformational (healing) experiences towards re-traditionalization were expressed by graduates of the treatment program.
					• These were categorized into 12 themes (in descending order): Feeling cared for, spiritual experiences, insight, making a commitment, empowerment, releasing emotional pain, remorse, reconnecting to traditional values, forgiveness, relief, safety, and gratitude.
Gossage et al, 2003 [46]	√	√	√	√	• IPsFU (Inmate/patients followed-up) drank 1 to 1.5 drinks less per drinking occasion than before intake (5.4 vs. 6.8), although still considered to be problematic.
					• Analysis using the Wilks test reveals significant improvements in scores over 3 time periods (baseline, 3, 9 months after release) for relating to the animal world and human world (p < 0.02 and p < 0.03) respectively.
					• Mean social support given to IP by his family increased before going to jail and at follow-up (from 6.5 to 8.3).
					• One of five indicators of domestic violence (hit or throw things first, regardless of who started an argument) improved significantly from before going to jail to follow-up (*x*^2^ = 4.714, p = 0.030).
					• Medical status scores improved before to follow-up (5.8 to 7.8 on a 10-point scale) and this was statistically significant (paired *t*-test, =3.3.16, p = 0.003).
					• There was substantial and significant improvement in marital status (*x*^2^ = 108.127, 45 df, p = 0.000).
					• 47% of IPs were rearrested at some point during the study.
Lowe et al, 2012 [48]			√	√	• Culturally based intervention (CTC) was significantly more effective for reducing substance use and related problems than the non-culturally-based intervention (SE) on the Global Assessment of Individual Needs—Quick (GAIN-Q) as follows:
					• The Total Symptom Severity Score (TSSS) showed differences between groups increased over time, and at 3 month follow-up, the difference remained and the magnitude increased (t = -5.35, p < .001).
					• The General Life Problem Index (GLPI) showed differences between the CTC and SE groups becoming significant at post intervention (t = -2.63, p = .009) and 3 month follow-up (t = -5.05, p < .001).
					• The Internal Behavior Scale (IBS) results show a significant difference between the two groups at post-intervention (t = -4.18, p < .001) and 3 month follow-up (t = -5.45, p < .001).
					• External Behavior Scale (EBS) score differences between the two groups became significant at post-intervention (t = -3.58, p < .001) and 3 month follow-up, (t = -4.56, p < .001).
					• The difference in the Substance Problem Scale (SPS) between the CTC and SE groups became significant at post-intervention (t = -3.89, p < .001) and 3 month follow-up, (t = -4.69, p = .001).
					• Cherokee self-reliance scores showed that at post-intervention, the CTC group had higher scores than the SE group (t = 2.72, p = .007). At 3 month follow-up, the difference between the two groups became larger (t = 6.74, p < .001).
Naquin et al, 2006 [49]				√	• Rate of residents completing the program rose dramatically from 2002-2005, from 55% in 2002 to 75% in 2005, and this level of retention is higher than the national experience of 35% for therapeutic communities and 33-38% for long-term care (over 30 days).
					• At 6 month follow-up, use of alcohol in the last 30 days dropped from 57% at intake to 20%.
					• Full-time employment increased from 19.2% to 33.3%.
Nebelkopf & Penagos, 2005 [50]			√	√	• Mixed results in self-reported quality of life results owing to population that included HIV/AIDS clients, e.g., “how would you rate your overall health” decreased between baseline and follow-up (no data provided) whereas “feeling bad lately” decreased over that period of time (32% vs. 3% said “definitely true”; 29% vs. 18% said “mostly true”).
Nebelkopf & Wright, 2011 [51]		√	√	√	• Using the McNemar test:
					• 24% reported using alcohol or drugs in the prior 30 days at baseline, with a decline to 5% six months later (p < .001).
					• Experiences of stress, emotion, or activities resulting from substance use in the prior 30 days also showed a decreasing rate of change from 47% to 23% (p < .001).
					• The number reporting either part or full-time employment increased from 11% to 20% (p < .001).
					• The largest rate of change was seen in enrollment in school or a training program, moving from 7% to 17% (p < .001).
					• The number reporting being arrested or committing a crime in the prior 30 days went from 31% to 5% (p < 0.001).
					• Significant reductions were seen in the rates of non-substance use-related reports of: serious depression (p < .001), serious anxiety or tension (p < .001), hallucinations (p < .001), trouble understanding or concentrating (p < .001), trouble controlling violent behavior (p < .01), and suicide attempts (p < .01).
Saylors, 2003 [52]			√	√	• Within pre/post matched sample, alcohol use decreased 13% after 6 months and drinking alcohol to intoxication was reduced by 19%.
					• Women who reported using other drugs at intake, such as marijuana and inhalants, reported no use at 6 months.
					• Heroin use was down 93%.
					• At 12 month follow-up, the rate of full-time employment increased from 10% at intake, to 29%, and the clients who were legally employed doubled.
					• There was an increase in the % of participants claiming good health and decreases of “fair” or “poor”.
					• Positive change in clients’ living situations also resulted in fewer having contact with the criminal justice system and more being enrolled in school or job training programs.
					• Culture was viewed as important at intake, with 5.7% reporting it was “not important”; 11.5% responding “important”, and 73% responding that their culture was “very important” to them.
Wright et al, 2011 [53]		√	√	√	• Using the McNemar test:
					• 80.2% decrease rate of change in alcohol and drug use from 116 (23.7%) in the prior 30 days at baseline to 23 (4.7%) six months later (p < .001).
					• Experiences of stress, emotion, or activities resulting from substance use in the prior 30 days showed a decreasing rate of change of 51.8%, from 231 (47.1%) to 111 (22.7%) (p < .001).
					• The number reporting either part or full-time employment increased from 55 (11.2%) to 100 (20.4%), with an 82.1% rate of change (p < .001).
					• The largest rate of change (150.7%) was seen in enrollment in school or a training program, moving from 34 (6.9%) to 85 (17.3%) (p < .001).
					• The number reporting being arrested or committing a crime (includes illegal substance use) in the prior 30 days went from 151 (30.8%) to 26 (5.3%) with an 82.8% rate of change (p < .001).
					• Significant reductions were seen in reports of serious depression (p < .001), serious anxiety or tension (p < .001), hallucinations (p < .001), trouble understanding or concentrating (p < .001), trouble controlling violent behavior (p < .01), and suicide attempts (p < .01).
	**Grey literature**				
Bresette, 2009/2010 [54]	√	√	√	√	Outcome #1: Increased sense of physical and mental well-being; feeling purpose and self-esteem:
					• Self-identity as a Native was much more positive at the end of treatment.
					• 71% of clients stated that they feel very comfortable practicing their cultural beliefs.
					• 93% of clients who entered the program did not have a spirit name and received one during their stay in the program.
					• 100% of clients stated that they were completely comfortable using their native language both in their community and outside their community.
					• 63% of clients stated that they had some connection to First Nations Culture, to family members or extended family.
					• 58% of clients returned to their community and participated in cultural, social or artistic activities in their home community.
					Outcome #2: Increased knowledge of drug-free lifestyles including cultural healing strategies:
					• Increased knowledge of drug-free lifestyles including cultural healing strategies, such as connection with spiritual family through youth fasting, feasts, ceremonies and learning to help self with use of the spirit.
					Outcome #3: More past clients pursued their education and/or life learning goals:
					• Average grade level improvement in language arts (.98% grade improvement) and math (.99% grade improvement).
					• Upon return to their communities, clients reported that they continue performing traditional cultural activities, e.g., smudging, leading prayer, assisting with dressing the drum, etc.
					• 100% of clients stated that they volunteer once a month in their community.
					• 33% stated when they call back to the Treatment Centre after discharge that they had increased their social activities.
					Outcome #4: Clients have developed positive social networks and have passed on teachings to help peers and community members:
					• 46% of the clients continued with culture either alone or with family, friends or community members.
					• Clients have connected with peers via the internet after leaving treatment.
					• Clients have identified a confidant (clients calling the NNHC on follow-up to treatment included 3 or 25% of the youth who were in the program within the last year. 15.2 hours total spent on the 24 hour, toll-free line with youth over 104 different contacts).
					Outcome #5: Clients encountered fewer occurrences with the justice system.
					• 37% of clients left treatment early, all were female and 60% left because of charges.
					• Serious occurrences (e.g., assaults on staff/clients) average = 1.5/month vs. 4.75 benchmark.
					• Incidents (e.g., physical attack/threats) average = 1.5/month vs. 6.3 benchmark.
D’Hondt, no year [55]			√	√	• High completion rate at 84.6% and 50.0% of patients continued to be engaged in aftercare programs at CAMH and elsewhere.
					• Reduced alcohol and drugs use in follow-up (30 days prior) compared to initial assessment (90 days prior).
					• Pre- and post-treatment results showed a decrease in BASIS 32 scores, suggesting clinically important improvements in general mental health and functioning among the clients.
					• At initial assessment (treatment entry), 10 out of 12 individuals (92%) reported having consumed alcohol to the point of blackout in the past 90 days. However, at follow-up, only 1 individual of the 9 contacted (11%) reported having drunk until blackout in the past 30 days.
Kunic, 2009 [56]				√	• Those who participated in Aboriginal Offender Substance Program (AOSAP) were returned to custody at a lower rate during the follow-up period than the groups of Aboriginal offenders who participated in National Substance Abuse Program- High Intensity (NSAP-H), NSAP-M (Moderate Intensity), failed to complete a substance use program, or did not participate in a substance use program prior to release from custody. Aboriginal offenders who participated in versions 2 or 3 of AOSAP were returned to custody at the same rate as Aboriginal offenders who participated in version 1 of AOSAP. There was no statistical difference between versions of AOSAP.
					• Only 5% of the successful participants of AOSAP- V 2&3, and 6% of the participants of AOSAP version 1 were returned to custody because of a new offence or charge compared to 16% and 20% of the successful participants of NSAP-H and NSAP-M, respectively.
					• Exposure to substance use treatment prior to release from custody was a relatively weak predictor of relapse to substance use (*p* = 0.07). However, some evidence suggested that successful participants of AOSAP and NSAP-M were less likely to incur a positive urinalysis result while on release than successful participants of NSAP-H.
					• Those who participated in AOSAP were less likely than offenders from the other program exposure categories to test positive for drugs that are considered dangerous (e.g., cocaine, opioids).
McConnery & Dumont, 2010 [57]	√	√	√	√	Outcome #1: Achieve greater balance in the four aspects of life (mental, spiritual, emotional, and physical):
					• Not a clear increase over time in all aspects of wellness; however:
					• Mental wellness of clients increased during treatment and 6 months after their treatment, but it was noted that there is “too much inaccuracy in the question to judge if there was a significant increase” (p.25).
					• The only marked finding under spiritual wellness was the increase of practice and comfort associated with practicing this type of spiritually during the program, such as the daily smudge and praying. The spiritual aspect was mentioned a few times in the Talking Circle as something that participants thought would help them to remain sober once they returned to their community. But it is noted that “the spiritual aspect does not show considerable changes that could be interpreted as a general increase for participants, despite the fact that they name this as an important tool for their recovery” (p. 25).
					• Authors noted: “The emotional aspect shows more clearly a decrease in feeling of sadness and crying” (p.25).
					• Definite increase in the self-interpretation of physical good health with time from treatment to 3 months after treatment. There is a slight decrease between 3 months after treatment and 6 months after treatment. The authors note that “in the physical aspect there is a more evident decrease in the feeling of ill health” (p.25).
					Outcome #2: Increase self-esteem and cultural pride:
					• Slight increase in self-esteem from 6 months prior to treatment and 6 months after treatment, but authors note this is not significant.
					• Cultural pride is about as high 6 months before treatment as it is 6 months after treatment.
					Outcome #3: Achieve abstinence and influence peers in communities.
					• 50% or more of the participants remained abstinent during the 6 months after treatment.
					Outcome #4: Decrease the number of occurrences of client-related family violence:
					• Slight decrease in violence from the pre-treatment to the post-treatment.
The Tsow Tun Le Lum Society [58]	√	√	√	√	Outcome #1: Clients are involved in more activities that contribute to their being “clean and sober” (at 3 months post treatment):
					• 2/3 (7 of 11) kept busy at daily activities every day or at least 3 times a week.
					• Staying in the company of sober people remained the same as upon admission at 45% (5 of 11).
					• 45% (5 of 11) requested help from AA/NA (a slight increase from admission).
					• 64% (7 of 11) put into practice new ways of reacting to risky situations.
					Outcome #2: Clients pride and dignity are empowered through participating in cultural, spiritual, and artistic events (at 3 months post treatment):
					• 55% (6 of 11) were comfortable self-identifying as Aboriginal or Inuit (this is a drop from that at admission of 82%).
					• 45% (5 of 11) had participated in cultural or traditional events (same as six months prior to admission).
					• None were uncomfortable with practicing Aboriginal spiritual practice.
					• 45% agreed or strongly agreed that a rich heritage of knowledge, wisdom, and traditional was passed to them (an increase over admission (36%) but a slight drop from the rate at completion (64%)).
					Outcome #3: A decrease in demonstration of violent behaviors towards self and others:
					• Significant drop in violent behaviors towards others, from 73% at admission to 29% at 3 months post treatment.
					• Self-violent behavior dropped from 27% at admission to 14% at 3 months post treatment.
					Outcome #4: Increased client’s self-esteem enhances their mental, physical, emotional, and spiritual well-being (at 3 months post treatment):
					• 36% prefer to use and stay in the company of people in recovery every day.
					• 36% have requested assistance from resources in their community (this % was double over the rate at admission).
					• 18% (2 of 11) had difficulty sleeping; 55% (6 of 11) could sleep without medication; and 64% (7 of 11) felt calm and rested from sleep (these % were improvements over rates at admission).
					Outcome #5: Increase awareness in communities around addictions and its impact on people:
					• Since leaving the Treatment Centre, clients most frequently got support from a friend or family member.

### Outcomes collected

Outcomes were collected on four main themes: Spiritual, Mental, Emotional, and Physical wellness with many positive results found in all areas. All but two articles (90%) focused on measuring physical wellness, which included five major subthemes ranging from improvement in physical health to sobriety or abstinence from alcohol, drug, or inhalant use. Emotional health was frequently collected in 74% of studies. It had nine subthemes ranging from self-esteem to non-violence or non-aggressive behaviour. Mental wellness was measured by just over half (53%) of the studies and was captured through knowledge, skills, and awareness; school achievement; and learning about Aboriginal spiritual healing. Fewer studies (37%) measured spiritual health, as identified via spiritual health practices, awareness and values; feeling connected/belonging; and traditional values practiced. Several studies (42%) focused on measuring dyad combinations of outcomes, particularly emotional and physical wellness. Just over one quarter (26%) of studies collected outcomes under all four themes.

## Main results

Results provide evidence about the benefits of Indigenous cultural interventions to help improve client functioning in all areas of wellness, particularly in association with reducing or eliminating substance use problems as found in almost three quarters (74%) of studies. It is important to note, however, that only two studies based on non-equivalent control groups, directly compared and contrasted the effects of Indigenous and Western components within the same study. Lowe et al. [48] found that a Native American adolescent culturally-based intervention was significantly more effective at reducing substance use and related problems than a non-cultural-based intervention. The largest significant differences between the groups for all four major scales of the Global Assessment of Individual Needs instrument occurred at the 3 month post-intervention follow-up. In contrast, Boyd-Ball [41] found no differences between treatment as usual (which in this setting also incorporated cultural activities), and family-enhanced intervention groups, both of which had 80-100% abstinence rates over 12 months. Mixed results were identified for self-reported quality of life, which Nebelkopf and Penagos [50] suggested are specific to the population that included HIV/AIDS clients. McConnery and Dumont [57] saw no clear increase over time in all aspects of clients’ wellness; however, there were meaningful changes in emotional and physical health.

## Discussion

This study set out to identify and describe what is known about the types of cultural interventions used with Indigenous populations to treat addictions, along with intended outcomes and effects on wellness in this context. We examined academic literature, but suggest that not all of the relevant evidence may be found through such sources, as much of the knowledge about culture is still held in Indigenous “worldviews, languages and rituals” [59]. All studies identified were from North America, and involved community-based, residential substance use treatment programs of varying lengths. They commonly integrated Indigenous and Western modalities of healing. A wide variety of cultural interventions were identified and, on average, at least half a dozen of these were offered at any given site. There was no pattern to the mixing or blending of cultural interventions or between cultural and Western interventions, suggesting that these were intentionally site specific. The rich diversity of the intervention components makes it difficult to compare the benefits of different modalities, especially across programs and settings.

Moreover, the complex and holistic approaches often used in cultural interventions complicates efforts to isolate and study specific components [51]. For example, the Aboriginal Offender Substance Abuse Program [56] incorporated a holistic model, which blends traditional healing with contemporary practices in addictions treatment. There are four modules in the intervention spanning 65 sessions within the program. The modules are based on the four directions that are fundamental to life and are embedded within Medicine Wheel teachings and Creation Stories. Using a traditional holistic healing approach implies an interconnectedness of the physical, spiritual, mental and emotional aspects of individual wellbeing. The mutuality of these relationships means that they are inseparable from one’s sense of personal/social responsibility and identity that exists within a collective society. Consequently, the holistic interventions and approaches to healing cannot be isolated into individual units of study and are best symbolized by a series of relationships depicted by concentric circles emanating from the self and encompassing the physical, emotional, spiritual and mental aspects of wellbeing within the four directions.

In addition, given the lack of implementation analyses in these studies, it is difficult to ascertain if positive or negative outcomes from a program are attributable to the intervention, or if these are reflective of issues with implementation. For example, organizational and infrastructure challenges, as well as issues with promotion or recruitment, may all impact intervention outcomes. Furthermore, in almost all integrative programs, client participation in designated cultural activities (especially traditional ceremonies) was voluntary; thus, in the absence of careful reporting, there is no way to ascertain the degree of client involvement in these optional activities or to estimate their impact on treatment outcomes.

Given the diversity of Indigenous people (there are over 630 First Nation governments or bands in Canada alone) and the manner in which cultural interventions are intimately tied to the Indigenous groups who developed and practice them, comparability and generalization across programs and settings remains an issue. One possible resolution to this challenge is to compare cultural interventions not so much on their distinctive forms (e.g., sweat lodge vs. shaking tent) but rather on their common functions (e.g., accessing traditional spirituality, enhancing cultural identity), with integration of these components into addictions treatment framed as events within complex dynamic systems [60]. Further conceptual work along these lines may overcome the problems of comparability and generalization in this domain. Beyond this issue, there were no controlled trials, and such methods may be incongruent with cultural values. Few studies included qualitative methodologies that might enhance understanding of outcomes, although this in part might be related to the inclusion criteria priorizing measured outcomes for treatment interventions. While, there was great variability in sample size, ranging from 11 to 2,685 participants, the majority of studies included under 100 participants. It is of note that studies with low *N*s might be useful as they are tailored to a very specific people at a very specific time and place.

The majority of studies conducted followed up shortly after treatment ended (post-treatment, or 3 months following treatment). It is possible that longer follow-up periods were not feasible given the dynamic nature of community priorities, high turnover in the treatment field, and movement of individual members (including intervention participants) to and from the community. Exceptions to this were studies that included a one year [42,52] or three year [51] follow-up period. It should be noted that these were residential treatment programs that had included a longer follow-up component as part of program delivery and study design. Further research is required to determine if outcomes observed in those studies with short term follow-up were sustained, and how best to maintain positive effects.

Ironically, despite the holistic and balanced nature of wellness to Indigenous people, few studies collected a holistic set of wellness outcomes. While most studies evaluated physical outcomes, such as sobriety, few studies explored spiritual outcomes such as feeling connected or having a sense of belonging. Only two studies included this as an outcome, both measuring comfort among participants in engaging in spiritual practice [57,58]. This is likely related to challenges in defining and measuring spiritual wellness. A systematic review by Monod et al. [61] described the different constructs and aims of 35 instruments used to assess spirituality in health care research. It highlighted diversity of spirituality constructs used in instrument development resulting in heterogeneity of measures. The authors noted the limited availability of instruments especially designed to measure current spiritual state and the paucity of data on the psychometric properties of most of these instruments. Only three instruments were found to have at least 50% of items that focus on patients’ current spiritual state and concluded that of these, the FACIT-Sp [62] and The Spirituality Index of Wellbeing [63] are regarded as the best ones to measure current spiritual state. The results also showed the lack of instruments to measure spiritual distress [61-63]. Clearly, more studies are required that explore meanings of spiritual wellness and that develop and test tools to capture changes in this dimension of health.

Finally, an analysis of how gender and culture interact to affect outcomes was not often addressed. While several studies did report outcomes for females and males separately [41,42,47,50,53,57,58], only three studies explored how gender influenced the outcomes of a particular cultural intervention. The study by Dell and colleagues [44] analyzed youths’ responses based on whether these were common to both females and males or gender-specific. Lowe and colleagues [48] discussed gender differences found in the data and the potential implications of this for substance use treatment programs. Finally, Naquin et al. [49] critiqued the lack of focus within the project on gender-specific characteristics and the implications for treatment. More studies are required that make the intersection of gender and culture and their influence on substance use and treatment outcomes explicit in the intervention design and analysis.

In summary, we found an array of Indigenous cultural interventions integrated within substance addiction treatment programs or as standalone interventions. These interventions addressed wellness in one or more of the four dimensions of wellbeing: Spirit, Physical- Behavioral, Mind- Mental, and Heart- Social and Emotional [34,38,39]. The measurement of outcomes of these interventions varied widely, yet these diverse approaches to measurement and the recognition of their cultural contexts together with other forms of evidence will serve to inform the work of the *Honouring Our Strengths: Culture as Intervention* project [34]. That project has the explicit aim to develop culturally-based instruments to meaningfully measure wellness arising from participation in cultural interventions offered in the context of addictions treatment for Indigenous people.

### Future directions

This scoping study has identified a corpus of research on the assessment of outcomes associated with traditional cultural interventions in the context of addiction treatment for Indigenous people. Given the common assertion in many Indigenous communities that “our culture is our treatment” [64,65], it is indeed promising that evaluation of this claim has commenced. It is important to recognize, however, that additional research is required to inform the postulated causal relationships between local cultural (or culturally-modified) interventions and treatment outcomes. In this respect, we offer five recommendations for future inquiry in this field.

First, given that most of the identified studies involve *integrative* treatment approaches, future investigators could more clearly describe the Indigenous cultural components of the programs under study, including details surrounding whether, how, and how often treatment clients participated in these throughout the study. Second, given that most of the identified studies used a wide range of outcome measures, future investigators could adopt measures representing three broad classes of outcome indicators, including standard assessments of substance use over time, surveys of Indigenous community-designated indicators of wellness or recovery, and qualitative perspectives on outcomes through the lens of the diverse people involved in treatment. Third, it will be important in future studies to more adequately describe and analyze how gender, age and other social determinants of health affect wellness outcomes.

Fourth, given that many of the identified studies did not involve a comparison group, future investigators, working in partnership with Indigenous communities, could ensure that outcomes are assessed under controlled conditions to ensure more robust evidence of treatment efficacy. Since most interventions of this nature are introduced at a community rather than at an individual level, additive, lagged time, or other non-randomized designs are most appealing. For example, a stepped wedge design may be considered in which all communities receive the intervention, but are randomized to receive them early or later. Finally, given that most of the identified studies involve a range of Indigenous cultural practices, future investigators could develop a taxonomy of such practices based on *function* rather than *form* to assist both with interpretation of findings from and adaptation of practices to diverse Indigenous communities (see Hawe et al. [60] for this type of recasting of the concept of intervention fidelity).

## Conclusions

Culturally-based substance treatment efforts for Indigenous people are diverse, drawing on a variety of Indigenous practices and traditions that circulated between and among distinctive Indigenous communities before the arrival of European settlers. These have been adopted and adapted by modern Indigenous communities for contemporary use alongside Western approaches and are purposively designed to be place-, person-, and time-specific to maximize their potential effectiveness. The evidence identified in this scoping study suggests that culturally-based interventions may be effective at improving functioning in all areas of wellness for Indigenous people in treatment for substance use problems and addictions.

On a practical level, the findings from this study may be useful to all treatment centres serving Indigenous people. Case managers and clinicians are encouraged to advocate for access to culture-based approaches in their work with Indigenous clients, should those clients desire such offerings. Working with management, organizational, and provincial decision-makers to support pathways for service delivery that includes access to culture-based services is essential in meeting the needs of Indigenous clients. This can be facilitated through careful collaboration with practitioners of culture-based approaches, and through partnership with Indigenous communities. Additionally, measuring outcomes must carefully consider appropriate cultural and contextual aspects of wellness and include key outcomes identified as important to Indigenous clients and communities seeking services.

The findings from this review are being used to inform a national study on the implementation and measurement of cultural interventions that support wellness in treatment centres serving Indigenous people in Canada. There is a need to develop valid and reliable culturally-based instruments or methods to meaningfully measure Indigenous wellness. Addiction researchers, treatment providers, and cultural knowledge holders are encouraged to work together to make further inroads into expanding the study of culturally-based interventions from multiple perspectives and locations, including sex/gender-based analysis. Finally, the authors found that the use of a Two-Eyed Seeing approach [33] which honors the strengths of both Indigenous and Western knowledges was useful for understanding and seeing the potential of the often integrated approaches used by treatment centres, and for co-learning by the research team.

### Endnotes

^a^In Canada, Indigenous aligns with the cultural names of First Nations, for example, Anishinabe or Haudenosaunee. Both mean people of the earth with meaning based in Creation stories that connect First Nations people to land, language, and nationhood. The United Nations [66] defines “Indigenous” as people who:

•Self-identify as Indigenous and are recognized and accepted by their community as a member

•Form non-dominant groups of society

•Resolve to maintain and reproduce their ancestral environments and systems as distinctive people and communities

•Demonstrate:

•Historical continuity with pre-colonial and/or pre-settler societies

•Strong links to territories and surrounding natural resources

•Distinct social, economic or political systems

•Distinct languages, cultures and beliefs.

## Competing interests

The authors declare that they have no competing interests.

## Authors’ contributions

BS helped design the study, participated in screening articles and helped to critically assess the paper. CD and CH conceived of the *Honouring Our Strengths: Culture as Intervention* project, participated in screening articles, and helped to critically review the paper. LH, CM, DM, JPG helped to screen articles, draft the manuscript and to revise the paper. MF participated in screening articles and organizing the references. MR helped design the study; acquired, organized and screened the articles; integrated the findings into tables and figures; led the writing of the manuscript and its revisions. NP conceived of the scoping study, participated in screening articles, helped draft the manuscript and to revise the paper. All authors are provided final approval of the version to be published.

## References

[B1] Schiff JW Moore K The impact of the sweat lodge ceremony on dimensions of well-being Am Indian Alsk Native Ment Health Res 2006 13 48 69 17602408 10.5820/aian.1303.2006.48

[B2] Schiff JW Pelech W The sweat lodge ceremony for spiritual healing J Relig Spiritual Soc Work Soc Thought 2007 26 71 93

[B3] McCabe G Mind, body, emotions and spirit: reaching to the ancestors for healing Couns Psychol Q 2008 21 143 152

[B4] Stone RAT Whitbeck LB Chen X Johnson K Olson DM Traditional practices, traditional spirituality, and alcohol cessation among American Indians J Stud Alcohol 2006 67 236 244 16562405 10.15288/jsa.2006.67.236

[B5] French LA Alcohol and other drug addictions among Native Americans Alcohol Treat Q 2004 22 81 91

[B6] Moore D Coyhis D The multicultural Wellbriety peer recovery support program: two decades of community-based recovery Alcohol Treat Q 2010 28 273 292

[B7] Walls ML Johnson KD Whitbeck LB Hoyt DR Mental health and substance abuse services preferences among American Indian people of the northern Midwest Community Ment Health J 2006 42 521 535 17143732 10.1007/s10597-006-9054-7 PMC1705498

[B8] National Native Alcohol and Drug Abuse Program (NNADAP)/National Youth Solvent Abuse Program (NYSAP) Treatment Centre Directory http://www.hc-sc.gc.ca/fniah-spnia/substan/ads/nnadap-pnlaada_dir-rep-eng.php

[B9] Dell CA Seguin M Hopkins C Tempier R Mehl-Madrona L Dell D Duncan R Mosier K From benzos to berries: treatment offered at an Aboriginal youth solvent abuse treatment centre relays the importance of culture Can J Psychiatry 2011 56 75 83 21333034 10.1177/070674371105600202

[B10] Szlemko WJ Wood JW Thurman PJ Native Americans and alcohol: past, present, and future J Gen Psychol 2006 133 435 451 17128961 10.3200/GENP.133.4.435-451

[B11] Hill DM Traditional Medicine in Contemporary Contexts: Protecting and Respecting Indigenous Knowledge and Medicine 2003 Ottawa: National Aboriginal Health Organization

[B12] Hill DM Traditional medicine and restoration of wellness strategies J Aboriginal Health 2009 5 26 42

[B13] Stewart S Indigenous helping and healing in counselor training Centre Native Pol Res Mon 2007 2 53 65

[B14] Hawkins EH Cummins LH Marlatt GA Preventing substance abuse in American Indian and Alaska Native youth: promising strategies for healthier communities Psychol Bull 2004 130 304 323 14979774 10.1037/0033-2909.130.2.304

[B15] Foxcroft D Ireland D Lowe G Breen R Primary prevention of alcohol misuse in young people Cochrane Database Syst Rev 2002 3 CD003024 12137668 10.1002/14651858.CD003024

[B16] Carson KV Brinn MP Labiszewski NA Peters M Chang AB Veale A Esterman AJ Smith BJ Interventions for tobacco use prevention in Indigenous youth Cochrane Database Syst Rev 2012 8 CD009325 22895988 10.1002/14651858.CD009325.pub2 PMC6486186

[B17] Ringwalt C Bliss K The cultural tailoring of a substance use prevention curriculum for American Indian youth J Drug Educ 2006 36 159 177 17153515 10.2190/369L-9JJ9-81FG-VUGV

[B18] Beach MC Gary TL Price EG Robinson K Gozu A Palacio A Smarth C Jenckes M Feuerstein C Bass EB Powe NR Cooper LA Improving health care quality for racial/ethnic minorities: a systematic review of the best evidence regarding provider and organization interventions BMC Public Health 2006 6 104 114 16635262 10.1186/1471-2458-6-104 PMC1525173

[B19] Gates S McCambridge J Smith LA Foxcroft D Interventions for prevention of drug use by young people delivered in non-school settings Cochrane Database Syst Rev 2006 1 CD005030 16437511 10.1002/14651858.CD005030.pub2 PMC13222737

[B20] Edwards C Giroux D Okamoto SK A review of the literature on Native Hawaiian youth and drug use: implications for research and practice J Ethn Subst Abuse 2010 9 153 172 20737343 10.1080/15332640.2010.500580 PMC2929928

[B21] Szapocznik J Lopez B Prado G Schwartz SJ Pantin H Outpatient drug abuse treatment for Hispanic adolescents Drug Alcohol Depend 2006 84 Suppl 1 S54 S63 16765535 10.1016/j.drugalcdep.2006.05.007

[B22] Webb MS Treating tobacco dependence among African Americans: a meta-analytic review Health Psychol 2008 27 Suppl 3 S271 S282 18979980 10.1037/0278-6133.27.3(suppl.).s271

[B23] MacLean SJ d'Abbs PH Petrol sniffing in Aboriginal communities: a review of interventions Drug Alcohol Rev 2002 21 65 72 12189006 10.1080/09595230220119345

[B24] Ivers RG A review of tobacco interventions for Indigenous Australians Aust N Z J Public Health 2003 27 294 299 14705285 10.1111/j.1467-842x.2003.tb00398.x

[B25] Taylor K Thompson S Davis R Delivering culturally appropriate residential rehabilitation for urban Indigenous Australians: a review of the challenges and opportunities Aust N Z J Public Health 2010 34 Suppl 1 S36 S40 20618291 10.1111/j.1753-6405.2010.00551.x

[B26] Clifford A Pulver LJ Richmond R Shakeshaft A Ivers R Smoking, nutrition, alcohol and physical activity interventions targeting Indigenous Australians: rigorous evaluations and new directions needed Aust N Z J Public Health 2011 35 38 46 21299699 10.1111/j.1753-6405.2010.00631.x

[B27] Bryant J Bonevski B Paul C McElduff P Attia J A systematic review and meta-analysis of the effectiveness of behavioural smoking cessation interventions in selected disadvantaged groups Addiction 2011 106 1568 1585 21489007 10.1111/j.1360-0443.2011.03467.x

[B28] Lu L Liu Y Zhu W Shi J Liu Y Ling W Kosten TR Traditional medicine in the treatment of drug addiction Am J Drug Alcohol Abuse 2009 35 1 11 19152199 10.1080/00952990802455469

[B29] Gone JP Alcántara C Identifying effective mental health interventions for American Indians and Alaska Natives: a review of the literature Cultur Divers Ethnic Minor Psychol 2007 13 356 363 17967104 10.1037/1099-9809.13.4.356

[B30] Brady M Culture in treatment, culture as treatment. A critical appraisal of developments in addictions programs for Indigenous North Americans and Australians Soc Sci Med 1995 41 1487 1498 8607039 10.1016/0277-9536(95)00055-c

[B31] Abbott PJ Traditional and western healing practices for alcoholism in American Indians and Alaska Natives Subst Use Misuse 1998 33 2605 2646 9818991 10.3109/10826089809059342

[B32] Greenfield BL Venner KL Review of substance use disorder treatment research in Indian country: future directions to strive toward health equity Am J Drug Alcohol Abuse 2012 38 483 492 22931083 10.3109/00952990.2012.702170

[B33] Iwama M Marshall M Marshall A Bartlett C Two-eyed seeing and the language of healing in community-based research Can J Native Educ 2009 32 3 23

[B34] Dell CA Honouring Our Strengths: Culture as Intervention in Addictions Treatment http://www.addictionresearchchair.ca/creating-knowledge/national/honouring-our-strengths-culture-as-intervention/

[B35] Arksey H O'Malley L Scoping studies: towards a methodological framework Int J Soc Res Meth 2005 8 19 32

[B36] Levac D Colquhoun H O’Brien KK Scoping studies: advancing the methodology Implement Sci 2010 5 59 67 20854677 10.1186/1748-5908-5-69 PMC2954944

[B37] Cooke A Smith D Booth A Beyond PICO: the SPIDER tool for qualitative evidence synthesis Qual Health Res 2012 22 1435 1443 22829486 10.1177/1049732312452938

[B38] Hopkins C Dumont J Cultural healing practice within National Native Alcohol and Drug Abuse Program/Youth Solvent Addiction Program services 2010 Canada: National Native Alcohol and Drug Abuse Program

[B39] Hopkins C Dumont J Deleary M Virgil T Phase I: Culture as Intervention Research 2012 Saskatoon: University of Saskatchewan

[B40] Anderson EN A healing place: ethnographic notes on a treatment center Alcohol Treat Q 1992 9 1 21

[B41] Boyd-Ball AJ A culturally responsive, family-enhanced intervention model Alcohol Clin Exp Res 2003 27 1356 1360 12966339 10.1097/01.ALC.0000080166.14054.7C

[B42] Boyd-Ball AJ Dishion TJ Myers MW Light J Predicting American Indian adolescent substance use trajectories following inpatient treatment J Ethn Subst Abuse 2011 10 181 201 21888498 10.1080/15332640.2011.600189 PMC4752201

[B43] Dell D Hopkins C Residential volatile substance misuse treatment for Indigenous youth in Canada Subst Use Misuse 2011 46 107 113 21609154 10.3109/10826084.2011.580225

[B44] Dell CA Chalmers D Bresette N Swain S Rankin D Hopkins C A healing space: the experiences of first nations and Inuit youth with equine-assisted learning (EAL) Child Youth Care Forum 2011 40 319 336

[B45] Edwards Y Cultural connection and transformation: substance abuse treatment at Friendship House J Psychoactive Drugs 2003 35 53 58 12733758 10.1080/02791072.2003.10399993

[B46] Gossage JP Barton L Foster L Etsitty L LoneTree C Leonard C May PA Sweat lodge ceremonies for jail-based treatment J Psychoactive Drugs 2003 35 33 42 12733756 10.1080/02791072.2003.10399991

[B47] D’Silva J Schillo BA Sandman NR Leonard TL Boyle RG Evaluation of a tailored approach for tobacco dependence treatment for American Indians Am J Health Promot 2011 25 Suppl5 S66 S69 21510789 10.4278/ajhp.100611-QUAN-180

[B48] Lowe J Liang H Riggs C Henson J Community partnership to affect substance abuse among Native American adolescents Am J Drug Alcohol Abuse 2012 38 450 455 22931079 10.3109/00952990.2012.694534 PMC3604899

[B49] Naquin V Trojan J O’Neil G Manson SM The Therapeutic Village of Care: an Alaskan Native alcohol treatment model Ther Communities 2006 27 107 123

[B50] Nebelkopf E Penagos M Holistic Native Network: integrated HIV/AIDS, substance abuse, and mental health services for Native Americans in San Francisco J Psychoactive Drugs 2005 37 257 264 16295008 10.1080/02791072.2005.10400517

[B51] Nebelkopf E Wright S Holistic system of care: a ten-year perspective J Psychoactive Drugs 2011 43 302 308 22400461 10.1080/02791072.2011.628922

[B52] Saylors K The women's circle comes full circle J Psychoactive Drugs 2003 35 59 62 12733759 10.1080/02791072.2003.10399994

[B53] Wright S Nebelkopf E King J Maas M Patel C Samuel S Holistic system of care: evidence of effectiveness Subst Use Misuse 2011 46 1420 1430 21810076 10.3109/10826084.2011.592438

[B54] Bressette N NNDAP and YSAP Impact Study Pilot Project 2010 Nimkee NupiGawagan: Healing Centre Inc

[B55] D’Hondt J Aboriginal community engagement at the Centre for Addiction and Mental Health: Developing partnerships in a residential substance abuse treatment cycle Toronto, Ontario: Centre for Addiction and Mental Health

[B56] Kunic D Varis DD The Aboriginal Offender Substance Abuse Program (AOSAP): Examining the Effects of Successful Completion on Post-release Outcomes 2009 Ottawa: Correctional Service of Canada

[B57] McConnery J Dumont P Report on Impact Study Pilot Project 2010 Maniwaki, Quebec: Wanaki Center

[B58] Tsow Tun Le Lum Society NNADAP and YSAP Impact Study Pilot Projection

[B59] Henderson JY First Nations legal inheritances in Canada: the Mikmaq model Man LJ 1995 23 1 31

[B60] Hawe P Shiell A Riley T Theorising interventions as events in systems Am J Community Psychol 2009 43 267 276 19390961 10.1007/s10464-009-9229-9

[B61] Monod S Brennan M Rochat E Martin E Rochat S Büla CJ Instruments measuring spirituality in clinical research: a systematic review J Gen Intern Med 2011 26 1345 1357 21725695 10.1007/s11606-011-1769-7 PMC3208480

[B62] Brady MJ Peterman AH Fitchett G Mo M Cella D A case for including spirituality in quality of life measurement in oncology Psychooncology 1999 8 417 428 10559801 10.1002/(sici)1099-1611(199909/10)8:5<417::aid-pon398>3.0.co;2-4

[B63] Daaleman TP Frey BB Wallace D Studenski SA The spirituality index of well-being: development and testing of a new measure J Fam Pract 2002 51 952 959 12485549

[B64] Gone JP Redressing first nations historical trauma: theorizing mechanisms for Indigenous culture as mental health treatment Transcult Psychiatr 2013 50 683 706 10.1177/1363461513487669 23715822

[B65] Gone JP Calf Looking PE American Indian culture as substance abuse treatment: pursuing evidence for a local intervention J Psychoactive Drugs 2011 43 291 296 22400459 10.1080/02791072.2011.628915

[B66] United Nations Permanent Forum on Indigenous Issues http://www.un.org/esa/socdev/unpfii/documents/5session_factsheet1.pdf

